# Robotic rectal resection: oncologic outcomes

**DOI:** 10.1007/s13304-020-00911-6

**Published:** 2020-11-10

**Authors:** Claudio Fiorillo, Giuseppe Quero, Roberta Menghi, Caterina Cina, Vito Laterza, Davide De Sio, Fabio Longo, Sergio Alfieri

**Affiliations:** 1 Digestive Surgery Unit, Fondazione Policlinico Universitario “A. Gemelli” IRCCS di Roma, Largo Agostino Gemelli, 8, 00168 Rome, Italy; 2 Università Cattolica del Sacro Cuore di Roma, Rome, Italy

**Keywords:** Robotic surgery, Rectal cancer, Long-term outcomes, Oncological outcomes

## Abstract

Robotic surgery has progressively gained popularity in the treatment of rectal cancer. However, only a few studies on its oncologic effectiveness are currently present, with contrasting results. The purpose of this study is to report a single surgeon’s experience on robotic rectal resection (RRR) for cancer, focusing on the analysis of oncologic outcomes, both in terms of pathological features and long-term results. One-hundred and twenty-two consecutive patients who underwent RRR for rectal cancer from January 2013 to December 2019 were retrospectively enrolled. Patients’ characteristics and perioperative outcomes were collected. The analyzed oncologic outcomes were pathological features [distal (DM), circumferential margin (CRM) status and quality of mesorectal excision (TME)] and long-term outcomes [overall (OS) and disease-free survival (DFS)]. The mean operative time was 275 (± 60.5) minutes. Conversion rate was 6.6%. Complications occurred in 27 cases (22.1%) and reoperation was needed in 2 patients (1.5%). The median follow-up was 30.5 (5.9–86.1) months. None presented DM positivity. CRM positivity was 2.5% (2 cases) while a complete TME was reached in 94.3% of cases (115 patients). Recurrence rate was 5.7% (2 local, 4 distant and 1 local plus distant tumor relapse). OS and DFS were 90.7% and 83%, respectively. At the multivariate analysis, both CRM positivity and near complete/incomplete TME were recognized as negative prognostic factors for OS and DFS. Under appropriate logistic and operative conditions, robotic surgery for rectal cancer proves to be oncologically effective, with adequate pathological results and long-term outcomes. It also offers acceptable peri-operative outcomes, further confirming the safety and feasibility of the technique.

## Introduction

With the progressive improvement of technology, there has been a gradual shift toward the use of minimally invasive approaches even for challenging procedures such as rectal resection [1, 2]. However, many concerns recently arose about the oncological efficacy of minimally invasive techniques [3]. For instance, oncologic outcomes after rectal resection are highly dependent on the appropriateness of the surgical procedure, with a well-established role of partial mesorectal excision (PME) for upper rectum tumors and total mesorectal excision (TME) for mid-low rectum lesions [4]. The quality of TME as well as the involvement of circumferential resection margins (CRM) are notably associated with long-term survival and local recurrence [5].

Although laparoscopic surgery has progressively gained popularity in rectal cancer treatment, its oncological efficacy has been put into question by multiple studies [6–8]. These results find justification in the demanding learning curve for the laparoscopic approach and in the case volume of the operating surgeon [9–11], as well as in the technical difficulty of laparoscopy in achieving an appropriate pelvic dissection due to non-articulating instruments, fulcrum effect and 2D-vision. 

The more recent introduction of the robotic platform was specifically aimed to overcome these limitations, thanks to high-quality three-dimensional imaging, favorable ergonomics, and high degrees of freedom of movement, that permit a more precise and comfortable dissection. Although multiple studies already demonstrated the safety and feasibility of robotic rectal resection (RRR), with a lower conversion rate and estimated blood loss [12–14], few and contrasting data are currently present on its oncological and long-term outcomes. In this respect, several case–control series and a small, single-center, randomized trial reported advantages in terms of TME quality and acceptable overall (OS) and disease-free survival (DFS) when the robotic technique was employed [13, 15–17]. Conversely, the ROLARR trial, that compared RRR and laparoscopic rectal resection (LRR), documented comparable results in terms of TME quality and CRM positivity between the two techniques [18].

This extreme heterogeneity of results find justification in the small populations and short follow-up of most retrospective case series as well as in the involvement of surgeons in different phases of the learning curve in the only trial reported in the literature [19].

This inevitably underlines the need for performing challenging procedures such as RRR in high volume centers by surgeons with proven experience, to obtain conclusive results on both clinical and oncological outcomes of the robotic approach.

With the aim of giving our contribution to this ongoing debate, we here present a single-surgeon’s experience of RRR in a tertiary referral center for the surgical treatment of rectal cancer, with particular focus on perioperative, pathological and oncologic outcomes.

## Methods

After Institutional Review Board (IRB) approval, all patients aged 18 years and older who underwent RRR at the Digestive Surgery Unit of the Fondazione Policlinico Universitario “A. Gemelli” IRCCS of Rome from January 2013 to December 2019, using the da Vinci robotic platform (Intuitive Surgical, Inc, Sunnyvale, CA) were retrospectively included in the study from a prospectively maintained database. Surgical treatment allocation to open, laparoscopic or robot-assisted approach was based on the surgeon’s experience and robotic platform availability. Open procedures were performed by the same surgeon with an extensive experience in open rectal resection, while the minimally invasive approach was reserved to all patients operated by S.A. At the time of the study, S.A. already performed at least 60 laparoscopic and 25 robot-assisted rectal resections, required to complete the learning curve for an adequate minimally invasive treatment of rectal cancer [20, 21]. The allocation to the laparoscopic or robot-assisted approach was dependent on the robotic platform availability (twice a month in the first 2 years and once a month in the remaining period). Exclusion criteria for the minimally invasive approaches were severe cardiovascular comorbidities, low pulmonary compliance, severe coagulopathy, previous multiple abdominal surgeries. The da Vinci Si platform was used until November 2014, while the Xi platform was employed in the remaining cases. Only patients affected by rectal adenocarcinomas within 15 cm from the anal verge were included in the series.

### Data collection

Clinic-demographic data (age, sex, the American Society of Anesthesiologists (ASA) score, tumor location and neoadjuvant treatment), intraoperative data (docking time, operative time, intraoperative complications and conversion rate) and post-operative outcomes (length of hospital stay, post-operative complications, reoperation rate, and post-operative mortality within 30 and 90 days after surgery) were retrospectively collected.

Tumor location was classified into the high rectum (10–15 cm from the anal verge), middle rectum (5–10 cm from the anal verge) and low rectum (≤ 5 cm from the anal verge).

Intraoperative complications were defined as any deviation from the ideal intraoperative course, namely, cardiopulmonary adverse events, anesthetic deviation from the normal course, unintended structural damage, massive hemorrhage [22].

Post-operative complications were defined as any deviation from the conventional post-operative course, and classified according to the Clavien–Dindo classification [23]. Complications classified as Clavien–Dindo ≥ 3 (3–4) were defined as those complication requiring surgical, endoscopic or radiologic intervention (Grade 3), or life-threatening complications requiring Intensive Care Unit (ICU) management (Grade 4).

Histopathological evaluation and staging were performed according to the TNM classification (AJCC Cancer Staging System, 8^th^ edition).

The analyzed oncologic features comprised both pathological characteristics and long-term outcomes.

Pathological characteristics included the evaluation of the distal margin (DM), the CRM and the TME completeness. Both the DM and CRM were defined as positive when ≤ 1 mm was evidenced between the tumor and the cut edge [7, 8]. Completeness of TME was defined as “complete”, “nearly complete” or “incomplete” [7]. TME was defined “complete” when the surface of the mesorectum did not present any defect; “nearly complete” in case of 1 or 2 areas of violation < 5 mm; “incomplete” when the specimen did not meet the above-mentioned criteria. Surgery was considered successful in the presence of all the 3 following parameters: DM > 1 mm, CRM > 1 mm, and “complete”/ “near complete” TME [7].

Long-term outcomes analysis comprised the local and distant recurrence rates, overall survival (OS) and disease-free survival (DFS).

### Preoperative staging and selection for neoadjuvant treatment

All patients underwent a colonoscopy, a thoraco-abdominal computed tomography (CT) scan and a pelvic magnetic resonance imaging (MRI) for preoperative staging. All cases were discussed at the multidisciplinary board meeting and neoadjuvant treatment (namely, chemotherapy, short-course radiotherapy or long-course chemoradiotherapy) was offered in case of radiological node-positive or extramural disease. In these patients, all radiological studies were repeated before surgery to evaluate tumor response. In the case of intraperitoneal cT3N0 tumors or cT3N0 tumors located at the peritoneal reflection, surgery was the first treatment of choice.

### Surgical technique

A mechanical bowel preparation was performed two days before surgery in all cases. Two g of cefazolin and 500 mg of metronidazole were administered at the anesthetic induction as antibiotic prophylaxis. The operative technique was previously reported [13]. Briefly, patients were placed in Trendelenburg position with a 15° angle of right tilt. Five trocars were placed in all cases allowing to perform a single docking procedure in all patients.

Surgical steps consisted in:

- Incision of the pelvic peritoneum and identification of the left ureter, gonadic vessels and hypogastric nerves;
- Medial to lateral dissection between the Gerota’s and Toldt’s fascia and ligation of the inferior mesenteric artery at its origin (1–2 cm from the aorta) and the inferior mesenteric vein at the inferior border of pancreas;
- In all cases of anterior rectal resection (ARR), the splenic flexure was mobilized, and the lesser sac was firstly entered through the inferior border of the pancreas, and subsequently from above through the separation of the omentum from the transverse colon;
- Initial mesorectal mobilization through a posterior dissection following the “Holy Plane” of the hypogastric nerves up to the region below the tumor site, and subsequent lateral and anterior dissection. A PME was performed up to 5 cm below the tumor in case of upper rectum tumors, while a dissection 1 cm below the lesion with TME was carried out in case of middle and low rectal tumors.

A Knight-Griffen anastomosis was performed in all cases, except for very low rectal tumors with a good response to neoadjuvant treatment. In these patients, a colo-anal anastomosis (CAA) was the first surgical option. A diverting ileostomy was performed in all cases of TME or in case of hydropneumatic test positivity after partial mesorectal excision.

In case of involvement of anal sphincters, an abdominoperineal resection (APR) was performed. The TME was carried out robotically, while the levator muscles resection was conducted perineally. A permanent extraperitoneal colostomy was then performed in all cases.

### Follow-up

Adjuvant chemotherapy or radiochemotherapy were administered on the base of the histopathological staging. All patients were followed-up every 6 months after surgery for the first year and then every 12 months. At each visit, patients underwent physical examination with rectal exploration, abdominal ultrasound and chest X-ray. The carcinoembryonic antigen testing was performed at each visit. A complete colonoscopy, thoraco-abdominal CT scan and pelvic MRI were prescribed every year for the first 5 years.

### Study outcomes

The primary endpoint of the study was the evaluation of the oncologic outcomes in terms of resection margins (DM, CRM and TME completeness), local and distant recurrence rate, OS and DFS.

Secondary endpoint was the evaluation of the above-mentioned perioperative outcomes.

As a further analysis, an evaluation of prognostic factors affecting OS and DFS was additionally performed.

### Statistical analysis

Continuous data were reported as means and standard deviation (± SD) or median values and range (min–max), while all categorical data were reported as numbers and percentages. Univariate analysis included Mann–Whitney U test, Student’s *t* test, *χ*^2^ test, and Fisher’s exact test. All tests were 2-tailed, and *p* ≤ 0.05 was considered statistically significant. OS and DFS were calculated using Kaplan–Meier curves. Univariate analysis was conducted to identify potential factors influencing OS and DFS. Significant variables at the univariate analysis were entered into a Cox proportional hazard model to identify independent predictors of OS and DFS. Results were expressed as odds ratio (OR) with 95% confidence intervals (CI). Data were analyzed using IBM SPSS Statistics for Windows, version 25 (SPSS Inc, Chicago, IL).

## Results

**Fig. 1:**
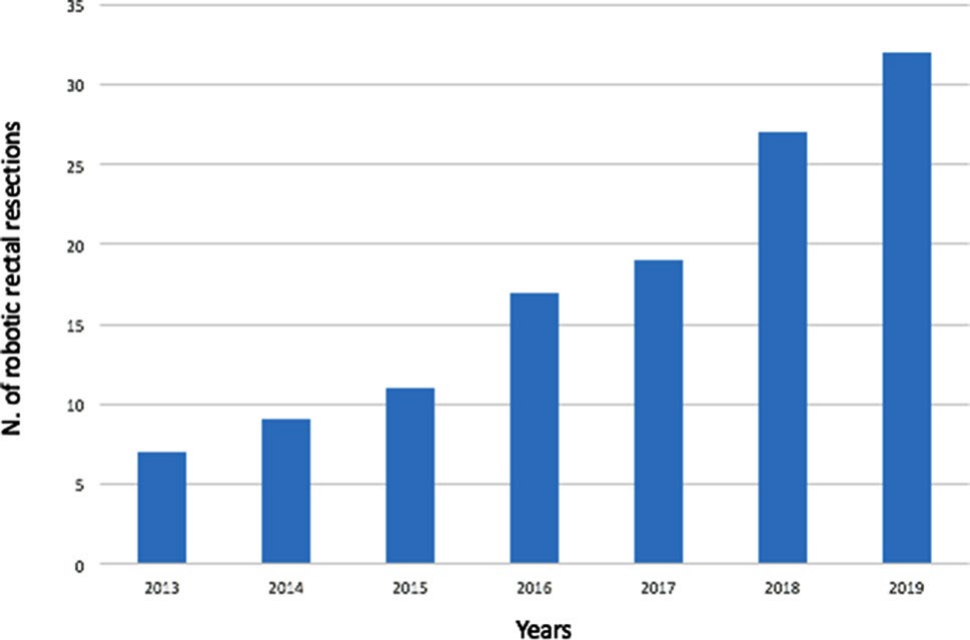
Number of robot-assisted rectal resection per year

During the study period, a total of 397 patients underwent rectal resection for rectal cancer. Of these, 234 were performed through a minimally invasive access: 112 laparoscopically and 122 robotically. All RRRs were included in the present study. RRRs were performed by the same surgeon through all the study period (S.A.), with a progressively annual increase of cases performed robotically (Fig. 1). Median follow-up of the study population was 30.5 (5.9–86.1) months. Clinic-demographic characteristics of the study cohort are reported in Table 1. The majority of patients presented tumors located in the middle [54 (44.3%)] or low rectum [39 (32%]. Sixty-five (53.3%) tumors were clinically staged as T3/T4 lesions, while node-positive disease was evidenced in 62 (50.8%) cases. Seventy-three (59.8%) patients underwent neoadjuvant treatment, which consisted in a long-course radiochemotherapy in most cases [61 (83.6%)]. The mean time interval between the end of the neoadjuvant treatment and surgery was 9.2 (± 2.1) weeks.

**Table 1:** Demographic and clinical patients’ characteristics

Total cases, *n*	122
Sex, *n* (%)	
Male	74 (60.7)
Female	48 (39.3)
Age, mean (± SD), years	63 (± 10.1)
BMI, *n* (%)	
18.5 < BMI < 24.9	45 (36.9)
25 < BMI < 30	51 (41.8)
BMI > 30	26 (21.3)
ASA score, *n* (%)	
ASA 1	40 (32.8)
ASA2	60 (49.2)
ASA 3	17 (13.9)
ASA4	5 (4.1)
Pre-operative clinical staging, *n* (%)	
cT1N0	14 (11.5)
cT1N1	9 (7.4)
cT2N0	18 (14.7)
cT2N1	9 (7.3)
cT2N2	7 (5.7)
cT3N0	20 (16.4)
cT3N1	17 (13.9)
cT3N2	15 (12.3)
cT4N0	8 (6.5)
cT4N1	4 (3.3)
cT4N2	1 (1)
Location of the tumor in the rectum, *n* (%)	
High	29 (23.8)
Middle	54 (44.3)
Low	39 (32)
Neoadjuvant therapy, *n* (%)	73 (59.8)
Long-course radiochemotherapy	61
Short-course radiotherapy	11
Chemotherapy alone	1
Previous abdominal surgery, *n* (%)	22 (18)

### Intra- and post-operative outcomes (Table 2)

**Table 2:** Intra- and post-operative outcomes

Operative time (min), mean (± SD)	275 (± 60.5)
Docking time (min), mean (± SD)	19 (± 11)
ARR	103 (84.4)
APR	19 (15.6)
Type of anastomosis, *n* (%)	
CRA	92 (75.4)
CAA	11 (9)
Diverting ostomy, *n* (%)	70 (57)
Temporary	51
Definitive	19
EBL, mean (± SD)	121 (± 92)
Conversion, *n* (%)	8 (6.6)
Morbidity, *n* (%)	27 (22.1)
Clavien-Dindo 1–2	16
Clavien-Dindo 3–4	11
Reoperation, n (%)	2 (1.5)
Length of hospital stay (days), mean (± SD)	9 (± 7)
30-day mortality, *n* (%)	3 (2.4)

*ARR* anterior rectal resection, *APR* abdomino-perineal resection, *CRA* colo-ractal anastomosis, *CAA* colo-anal anastomosis, *EBL* estimated blood loss

**Fig. 2:**
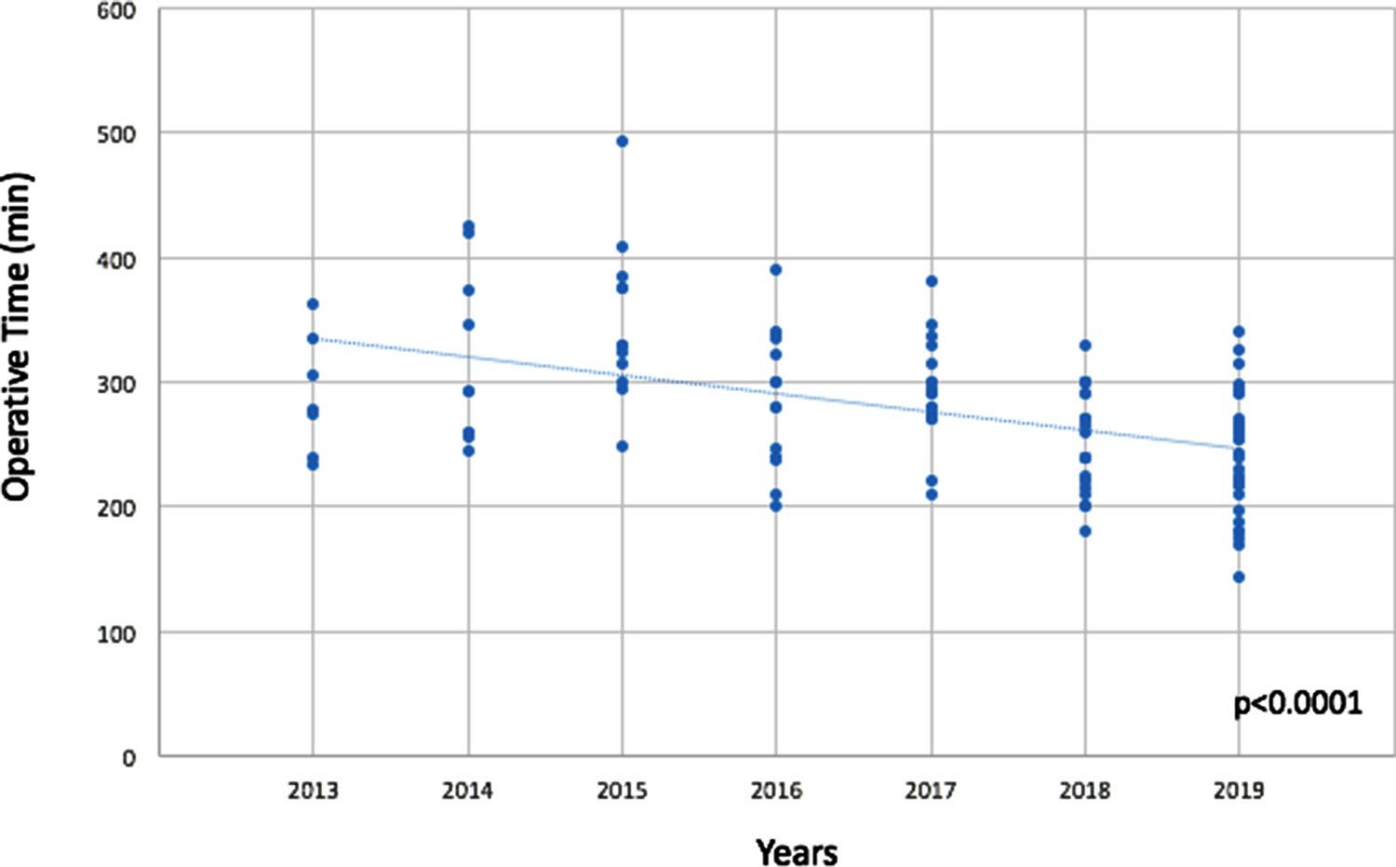
Operative time over the study period

The mean operative time was 275 (± 60.5) minutes, with a mean docking time of 19 (± 11) minutes. Operative time significantly decreased over the study period [323.9 (± 66.99) minutes in the years 2013–2015 vs 281.5 (± 47.8) minutes in 2016–2017 vs 249.7 (± 45.3) minutes in 2018–2019; *p* < 0.0001; Fig. 2]. The majority of patients underwent a spincter-preserving procedure [103 (84.4%)], with a colorectal anastomosis in 75.4% of cases (92 patients) and a coloanal anastomosis in 9% of cases (11 patients). Conversion to open surgery was needed in 8 patients (6.6%), due to excessive visceral fat that made dissection and retraction impossible in 3 cases, intraoperative bleeding in 3 patients and excessive adhesions due to previous surgeries in the remaining 2 cases. There was no conversion from robotic to laparoscopic approach. The mean length of hospitalization was 9 (± 7) days Severe post-operative complications rate (Clavien–Dindo grade ≥ 3) was 9% (11 patients). The most frequent major complication was anastomotic leakage with a rate of 5.7% (7 patients), requiring reoperation in 2 cases on post-operative day 5 and 6, respectively. In both cases, a laparotomy with peritoneal toilette and diverting ostomies was performed. The whole 30-day post-operative mortality rate was 2.4% (3 patients), due to myocardial infarction in an ASA 3 patient, and sepsis due to anastomotic leakage and *ab ingestis* pneumonia in the remaining 2 cases, respectively. No mortality was registered at 90 days from surgery.

### Oncologic outcomes: pathological features and long-term outcomes

Histological data and long-term outcomes are reported in Table 3. The TME quality assessment evidenced a complete excision in 94.3% of cases (115 patients), while a near-complete and incomplete excision were found in the 4.1% (5 patients) and 1.6% (2 patients) of cases, respectively. CRM was negative in 97.5% of the resections (119 patients). The three cases with positive CRM presented a microscopic margin infiltration and underwent adjuvant chemotherapy. No distal margin positivity was documented in any case. According to the Z6051 criteria [7], a successful resection was achieved in 120 out of 122 cases (98.3%).

**Table 3:** Pathological characteristics and long-term outcomes

Quality of mesorectal excision, *n* (%)	
Complete	115 (94.3)
Near complete	5 (4.1)
Incomplete	2 (1.6)
CRM negative, *n* (%)	119 (97.5)
Distal margin negative, *n* (%)	122 (100)
TMN stage, *n* (%)	
0	18 (14.8)
I	36 (29.5)
IIa	31 (25.4)
IIb	4 (3.3)
IIIa	12 (9.8)
IIIb	12 (9.8)
IIIc	9 (7.4)
T stage	
Tis	10 (8.2)
T0	9 (7.4)
T1	28 (23)
T2	29 (23.7)
T3	36 (29.5)
T4	10 (8.2)
N stage	
N0	89 (72.9)
N1	28 (23)
N2	5 (4.1)
Tumor dimension (cm), mean (± SD)	2.6 (± 1.6)
Lymph nodes harvested, mean (± SD)	11 (± 5.3)
Follow-up, months mean (± SD)	32 (± 22.5)
Adjuvant treatment, *n* (%)	52 (42.6)
Recurrence, *n* (%)	7 (5.8)
Local	2
Locale + distant	1
Distant	4
Mortality at follow up, *n* (%)	5 (4.1)

**Fig. 3:**
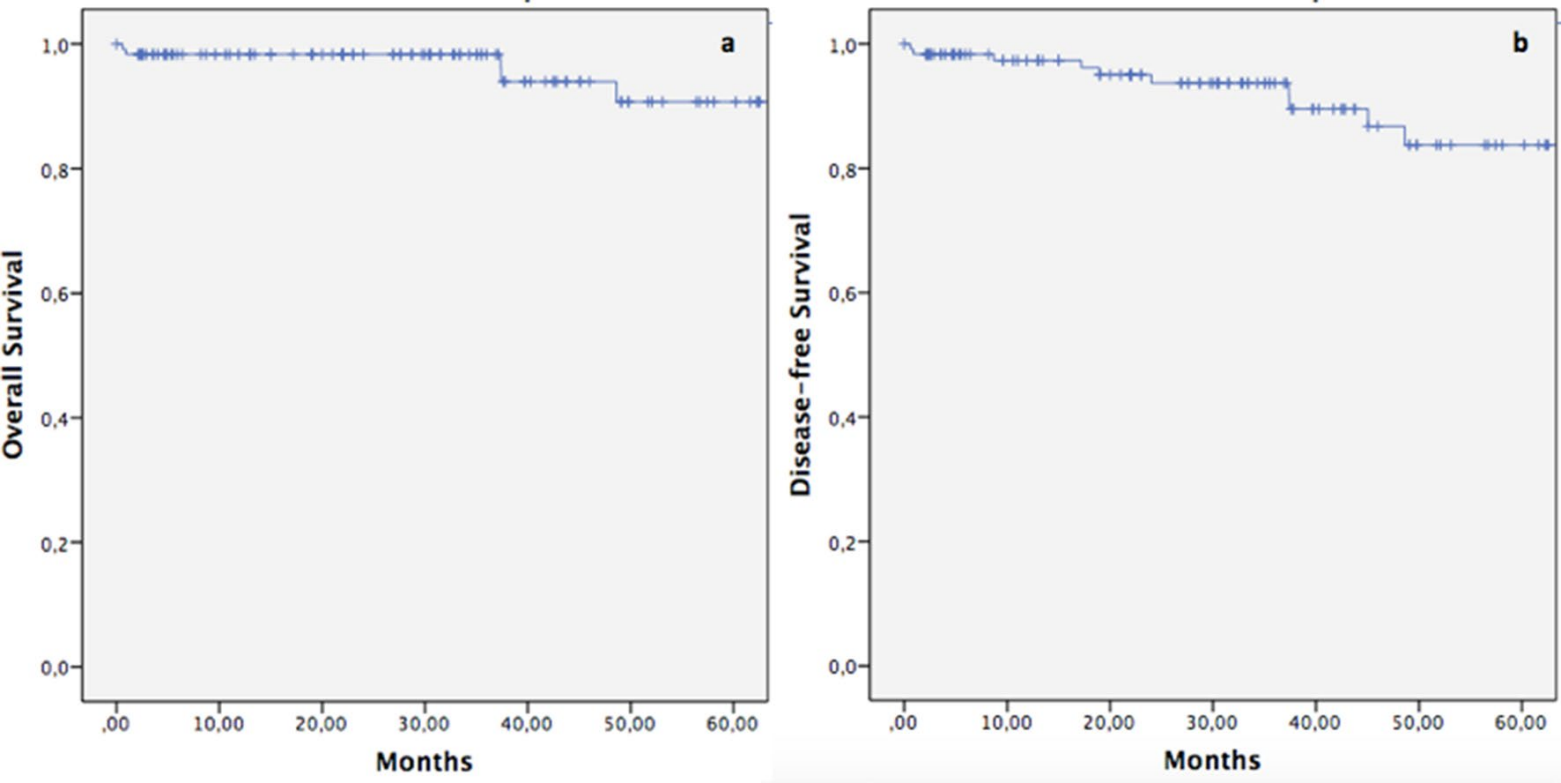
5-year overall and disease-free survival of the study population

Follow-up was completed in 121 out of 122 patients. A total of 7 patients (5.8%) developed tumor recurrence during the follow-up. Two patients with CRM positivity had local recurrence, while 1 patient developed local recurrence and liver metastases. Distant tumor relapse was documented in 4 cases: 1 isolated pulmonary recurrence and 3 cases of liver metastases. The mean time between surgery and the development of local/distant recurrence was 18.2 (± 7.2) months. The whole mortality rate at the last follow-up was 4.1% (5 patients). Long-term outcomes analysis in terms of OS and DFS are shown in Fig. 3. Five-year estimated OS was 90.7%, while the 5-year estimated DFS was 83%.

### Prognostic factors affecting OS and DFS: multivariate analysis (Table 4)

**Table 4:** Univariate analysis and multivariate Cox regression analysis for OS and DFS

	Univariate analysis				Multivariate analysis					
Variable	5-year OS (%)	*p*	5-year DFS (%)	*p*	5-year OS			5-year DFS		
					OR	95% CI	*p*	OR	95% CI	*p*
Age, ≥ 65/ < 65	83/96	0.1	80.6/85.1	0.92	–	–	–	–	–	–
Sex, M/F	89.2/93.3	0.26	85.5/82.3	0.85	–	–	–	–	–	–
ASA, I–II/III–IV	100/89.2	0.33	85/69.6	0.37	–	–	–	–	–	–
BMI, < 25/ ≥ 25	95.5/80.8	0.6	87.8/76.4	0.57						
Tumor location, high-middle/low	91.4/87.6	0.5	85/77.5	0.47	–	–	–	–	–	–
Neadjuvant treatment, yes/no	92.8/88.8	0.7	80.1/87.9	0.35						
Type of surgery, ARR/APR	91.5/85.7	0.77	82.9/85.7	0.48	–	–	–	–	–	–
Diverting ostomy, yes/no	86.3/95	0.1	80/87.9	0.35	–	–	–	–	–	–
Conversion, yes/no	90/100	0.5	80/83.6	0.42						
Complications, yes/no	96.3/88.5	0.78	75.9/86	0.21	–	–	–	–	–	–
Reoperation, yes/no	90.5/100	0.73	83/100	0.61	–	–	–	–	–	–
TNM stage, I–II/III	96.1/71.6	0.04	94.6/51.6	< 0.0001	0.1	–0.3 to 0.6	0.47	1.2	0.47–2	0.02
Lymph nodes harvested^a^, ≤ 11/ > 11	92.6/86.6	0.3	83.5/82.4	0.23	–	–	–	–	–	–
CRM, positive/negative	33/95.7	< 0.0001	33.3/88	< 0.0001	7.6	6.3–9	< 0.0001	3.7	1.4–6.1	0.02
TME quality, complete/near complete-incomplete	98/21.4	< 0.0001	93.3/14.3	< 0.0001	1.8	1.34–2.25	< 0.0001	3.9	3.1–4.7	< 0.0001

^a^The mean values of lymph nodes harvested was used for the univariate and multivariate analysis

*OS* overall survival, *DFS* disease-free survival, *OR* odds ratio, *CI* confidence interval

A further evaluation of the prognostic factors affecting OS and DFS was additionally conducted. At the univariate analysis, OS was significantly influenced by stage III tumors (*p* = 0.04), CRM positivity (*p* < 0.0001) and a near-complete/incomplete TME excision (*p* < 0.0001). The same features were recognized as influencing factors on DFS at the univariate analysis (stage III tumors: *p* < 0.0001; CRM positivity: *p* < 0.0001; near-complete/incomplete TME: *p* < 0.0001).

At the multivariate analysis, only CRM positivity (OR: 7.6; 95% CI 6.3–9; *p* < 0.0001) and near -complete/incomplete TME (OR: 1.8; 95% CI 1.34–2.25; *p* < 0.0001) were confirmed as negative influencing factors on OS.

Conversely, DFS was confirmed as negatively influenced by stage III tumors (OR: 1.2; 95% CI 0.47–2; *p* = 0.02), CRM positivity (OR: 3.7; 95% CI 1.4–6.1; *p* = 0.02) and near-complete/incomplete TME (OR: 3.9; 95% CI 3.1–4.7; *p* < 0.0001).

## Discussion

We here reported a retrospective analysis of a single surgeon’s experience from a tertiary referral center on the robot-assisted treatment of rectal cancer, with the specific aim to evaluate the appropriateness of this minimally invasive approach in guarantying an oncologically correct treatment of rectal tumors. As a secondary aim, we further evaluated the safety and feasibility of the robot-assisted approach, giving an overview of the short-term outcomes after RRR. According to our results, RRR can be considered a safe and feasible procedure, with a very low rate of CRM positivity, a high rate of appropriate TME and good long-term outcomes in terms of OS and DFS.

However, to validate the robot-assisted oncologic effectiveness, a comparative analysis between the outcomes we achieved and the current evidences in the literature on both the laparoscopic and robot-assisted techniques for rectal cancer treatment is mandatory. In this last regard, the high expectation was initially set on conventional laparoscopy. However, two large randomized controlled trials [7, 8] failed to demonstrate non-inferiority in comparison to the open approach. More specifically, the ALaCaRT study reported a positive CRM in the 7% of the laparoscopic procedures as compared to 3% in the open arm. Similarly, a higher rate of CRM positivity was documented in the Z6051 trial for laparoscopic proctectomy, with a positive CRM rate up to 12% vs 8% in the open group. A further evaluation of the oncological appropriateness of laparoscopic proctectomy was also conducted by Kim et al. [24], who reported a R2 rate up to 30% for locally advanced rectal tumors.

Similarly, a high rate of incomplete TME was reported in both the ALaCaRT (2%) and Z6051 (6%) trials [7, 8], with a non-significant trend in favor of the open approach over conventional laparoscopy in both studies. As compared to these data, the results we achieved are significantly favorable, with a whole CRM positivity in only the 2.5% of cases. These outcomes do not significantly differ from most of the other case series on RRR reported in the literature. D’Annibale et al. [25] documented CRM positivity in none of the patients who underwent robot-assisted proctectomy as compared to the laparoscopic approach. Similar results were also achieved in one of the largest case series on RRR [26], where CRM positivity had an incidence of 2.6%. A further confirmation was also given by a comparative meta-analysis (robot-assisted vs laparoscopic rectal resection) conducted by Xiong et al. [27], which demonstrated a significant superiority of the robotic approach in achieving CRM negativity.

With regards to TME integrity, we documented a complete excision in 94.3% of cases. Although our results are in line with the experience already published by Baik et al. [28] and Baek et al. [29], the significant debate is still present in the literature on the superiority of the robotic approach over conventional laparoscopy for an appropriate TME. More specifically, the two above-mentioned comparative studies found a significant advantage of the robotic technique over the laparoscopic one. Conversely, a recent meta-analysis by Rausa et al. [30] found the two minimally invasive approaches equivalent for a complete mesorectal excision, although a non-statistically significant superiority was documented for the robot-assisted approach.

Despite these evidences apparently support the superiority of RRR over LRR for both CRM negativity and TME quality, a specific comparison of our results with the only randomized clinical trial comparing robotic and laparoscopic proctectomy [18] is mandatory. In this last study, CRM positivity was found in 5.1% of robotic procedures (significantly higher as compared to ours and most of the other case series on RRR), with no statistical difference in comparison to LRR (6.3%). Regarding the TME integrity evaluation, unfortunately, the use of a different classification does not allow an appropriate and valid comparison. Although the randomized design of the study was initially thought to give a significant contribution in defining the role of RRR, multiple drawbacks of the ROLARR trial should be underlined before drawing any conclusion. As we previously stated [19], the inhomogeneous experience of the authors in robotic surgery and the absence of blinding to treatment allocation may rise some skepticism on the results obtained.

In terms of long-term results, we found a 5-year OS and DFS of 90.7% and 83%, respectively, which are comparable to other case series on RRR and LRR [31–34]. Moreover, to specifically evaluate the influence of the surgical technique on these long-term outcomes, we also focused our analysis on both the local and distant recurrence rates. Notably, we found an incidence of local tumor relapse of 1.6% (2 patients), lower than the current evidences on LRR [32, 33] and comparable to other robot-assisted case series [32, 35].

In the light of these results we can, thus, speculate that the robotic approach is oncologically appropriate in the treatment of rectal cancer. This may find justification in the multiple advantages offered by the robotic platform in comparison to laparoscopy. The rigid instruments, the restricted dexterity and the two-dimensional imaging are notably the major limitations of laparoscopy. In addition, operating in a restricted field as the pelvic cavity represents a further amplification of the difficulties of the laparoscopic approach. The introduction of the robotic platform was specifically aimed to overcome these limitations, thanks to the high-definition, three-dimensional imaging and the seven degrees of wrist-like motion. This permits a better dissection and retraction and may justify the low rate of CRM positivity and incomplete TME, and low rate of local recurrence in our case series.

Independently of the type of minimally invasive approach used, it is however important to underline the key role played by an adequate learning curve of the surgeons and the case-volume of the treatment centers. Both these factors are fundamental features to achieve appropriate oncological outcomes, as already reported by previous experiences [9–11].

The same technical advantages guaranteed by the robotic platform, together with adequate surgical expertise in robotic surgery, inevitably reflect also in the already proven better short-term outcomes as compared to laparoscopy.

Our conversion rate was 6.6%, in line with previous case series publications [12, 36, 37], and lower than the 8% reported in the ROLARR trial [18], further supporting the superiority of the robotic approach over laparoscopy. In addition, intraoperative complications rate was 2.4% (3 intraoperative hemorrhages), notably lower than the ROLARR trial data for both the laparoscopic (14.8%) and robotic (15.3%) techniques. Similar advantages were also observed for the post-operative complication incidence. We evidenced a whole rate of 22.1% and, of note, less than half of the patients experienced a Clavien-Dindo grade 3–4. Even in this case, we achieved significantly better results as compared to the only trial data (33.1% and 31.7% for the robotic and laparoscopic groups, respectively) [18]. In this regard, we already reported, in a previous experience, advantages in terms short-term outcomes of the robot-assisted approach over laparoscopy [38].

To the authors’ knowledge, our series is the largest one on robotic rectal cancer treatment in Italy and one of the largest experiences in Europe [15, 25, 37, 39–44] (Table 5), further confirming the benefits already reported by the other large US and Eastern studies [26, 31]. This supports the clear advantages of the robotic platform over the other approaches when surgery is performed by experienced surgeons in tertiary referral centers, especially for challenging procedures such as proctectomy. Currently, the only important limitation to the routine use of the robotic platform is represented by its costs. We already published the economic implications of using the robotic technique for rectal cancer treatment [38], confirming the higher total costs of the robotic approach in comparison to conventional laparoscopy, as already reported by previous publications [29, 42]. However, the introduction in the near future of new/robotic platforms with competitive costs will hopefully lead to a major dissemination and, thus, routine use of the robotic approach for rectal resections.

**Table 5:** Literature review on European experiences in robotic rectal surgery

Author	Type of study	Year	City	Number of patients	Years of analysis
D’Annibale et al. [41]	Retrospective	2004	Padua	10 RRR	2001–2003
Bianchi et al. [39]	Retrospective	2010	Milan	25 RRR vs 25 LRR	2008–2009
D’Annibale et al. [25]	Retrospective	2013	Rome	50 RRR vs 50 LRR	2006–2012
Colombo et al. [40]	Retrospective	2015	France	60 RRR vs 60 LRR	2009–2013
Allemann et al. [15]	Retrospective	2015	Switzerland	20 RRR vs 46 LRR vs 7 ORR	2012–2014
Valverde et al. [37]	Retrospective	2016	France	65 RRR vs 65 LRR	2013–2016
Morelli et al. [42]	Retrospective	2016	Pisa	50 RRR vs 25 LRR	2009–2014
Spinelli et al. [44]	Retrospective	2017	Milan	12 RRR	2015–2016
Rouanet et al. [43]	Retrospective	2018	France	200 RRR vs 200 LRR	2008–2015

This study presents some limitations. For instance, its retrospective design and the absence of a laparoscopic group of comparison are the major drawbacks. However, the long follow-up we reported significantly contributed to obtaining an adequate long-term evaluation, both in terms of OS and DFS, and local and distant tumor relapse rates. These last data strongly support the adequateness of the robotic approach regarding oncologic outcomes. However, the need for randomized studies, involving only high skilled surgeons and with standardization of the operative technique, is undeniable. Thus, until any technique has proven its superiority over the others with strong evidence, patients should be referred to high volume centers regardless of the surgical approach chosen.
